# Thickness-induced metal to insulator transition in Ru nanosheets probed by photoemission spectroscopy: Effects of disorder and Coulomb interaction

**DOI:** 10.1038/s41598-020-58057-9

**Published:** 2020-01-30

**Authors:** Daiki Ootsuki, Kenjiro Kodera, Daiya Shimonaka, Masashi Arita, Hirofumi Namatame, Masaki Taniguchi, Makoto Minohara, Koji Horiba, Hiroshi Kumigashira, Eiji Ikenaga, Akira Yasui, Yoshiharu Uchimoto, Satoshi Toyoda, Masahito Morita, Katsutoshi Fukuda, Teppei Yoshida

**Affiliations:** 1 Graduate School of Human and Environmental Studies, Kyoto University, Sakyo-ku, Kyoto 606-8501 Japan; 2 Hiroshima Synchrotron Radiation Center, Hiroshima University, Higashi-hiroshima, 739-0046 Japan; 3 Institute of Materials Structure Science, High Energy Accelerator Research Organization (KEK), Tsukuba, Ibaraki 305-0801 Japan; 4 SPring-8/JASRI, 1-1-1 Koto, Sayo-cho, Hyogo 679-5198 Japan; 5 Department of Materials Science and Engineering, Kyoto University, Sakyo-ku, Kyoto 606-8501 Japan; 6 Office of Society-Academia Collaboration for Innovation, Kyoto University, Sakyo-ku, Kyoto 606-8501 Japan

**Keywords:** Materials science, Physics

## Abstract

We investigated the electronic structures of mono- and few-layered Ru nanosheets (*N* layers (L) with *N* = 1, ~6, and ~9) on Si substrate by ultra-violet and x-ray photoemission spectroscopies. The spectral density of states (DOS) near *E*_F_ of ~6 L and 1 L is suppressed as it approaches *E*_F_ in contrast to that of ~9 L, which is consistent with the Ru 3 *d* core-level shift indicating the reduction of the metallic conductivity. A power law *g*(*ε*) ∝ |*ε* − *ε*_*F*_|^*α*^ well reproduces the observed spectral DOS of ~6 L and 1 L. The evolution of the power factor *α* suggests that the transition from the metallic state of ~9 L to the 2-dimensional insulating state with the soft Coulomb gap of 1 L through the disordered 3-dimensional metallic state of ~6 L.

## Introduction

**Figure 1:**
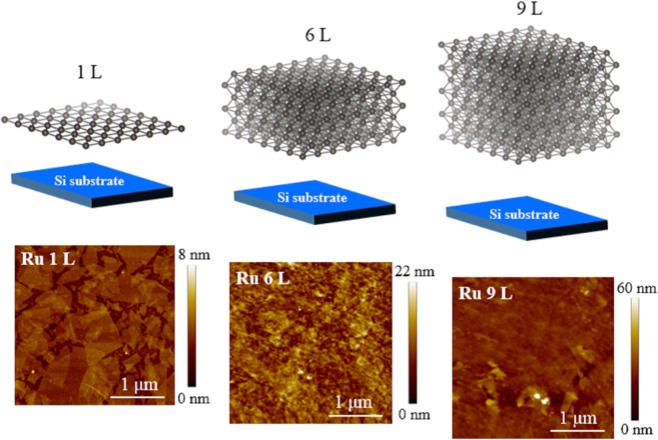
Schematics and AFM images of Ru nanosheets (1, 6, and 9 L).

The discovery of 2-dimensional crystal composed of a carbon monolayer, so called graphene, has evoked much attention to the physical properties in the atomic layer limit as well as applied researches for the electronic and photonic devices^1^. What is interesting about atomic layer crystals such as graphene, silicene, germanene, and stanenen is the different physical properties from the bulk nature^1–6^. Recently, 2-dimensional material Ru nanosheet consisting of a single element Ruthenium was successfully synthesized by topotactic reaction method^7^. The schematics of the mono- and few layered Ru nanosheets are displayed in Fig. 1. The optical absorption spectra of Ru nanosheets highly depend on the number of layers, indicating the disappearance of the metallic conductivity in mono-layered (1 L) and bi-layered Ru nanosheets (2 L)^8^. Toyoda *et al*. suggested that the ligancy-driven covalency and the metallicity abruptly occur between bilayer (2 L) and trilayer (3 L) Ru nanosheets^9^. Controlling the metallicity by the number of layers are promising applications associated with microelectronics. However, the mechanism responsible for the non-metallic behavior in the mono-layered Ru nanosheets is unknown.

In this work, we investigated how the electronic structure evolves from the mono- (1 L) to few layered Ru nanosheets ~6 and ~9 L on Si substrate by performing photoemission spectroscopy in order to clarify the mechanism of the disappearance of the metallic conductivity. We observed the chemical shifts of Ru 3 *d* core-level spectra and the suppression of the spectral weight at Fermi-level (*E*_F_) with decreasing the number of stacking layers. These indicate the disappearance of the metallic conductivity in the mono-layered Ru nanosheets. Moreover, we reproduced the spectral weight near *E*_F_ using a power law *g*(*ε*) ∝ |*ε* − *ε*_*F*_|^*α*^. The evolution of the power factor *α* suggests that the transition from the metallic state of ~9 L to the 2-dimensional insulating state with the soft Coulomb gap of 1 L through the disordered 3-dimensional metallic state of ~6 L.

## Methods

Ru nanosheets were prepared following the previously described an electrostatic self-assembly method in Refs. ^7,8^. A Si (111) wafer as substrate was cleaned by HCl-CH_3_ OH (1:1) solution and then by H_2_SO_4_ solution. The Si wafer was immersed in an aqueous solution of coblock polymer composed of polyvinyl alcohol and polyvinylamine for 20 min to coat the surface. It was then dipped in a colloidal suspension of negatively charged RuO_2_ nanosheets. The number of layers *N* was controlled by repeating this cycle. The obtained RuO_2_ nanosheets were reduced by the hydrogen reduction at 200 °C under the gas-flow-controlled condition (5% H_2_ and 95% Ar, 250 mL/min) and the Ru nanosheets were fabricated. The Ru nanosheets are composed of the atomic mono-layered hexagonal system with the bond length of the first-nearest-neighbor atoms to be 2.74 Å for 1 L and 2.65 Å for ≥6 L^7^.

A tapping-mode atomic force microscopy (AFM; Innova, Bruker Inc.) was used to obtain Ru nanosheets images. The AFM images of the fabricated Ru nanosheets for 1 L, ~6 L, and ~9 L are displayed in Fig. 1. The AFM image of 1 L shows single-crystal arrays, overlapped patches, and a bare surface. The total coverage is estimated to be approximately 82.5% and the fraction of the overlapping region to be 33.7%. For the few-layered Ru nanosheets, the number of layers is basically determined by the cycles of the fabrication process used to prepared the RuO_2_ nanosheets. In our fabrication process, the few-layered Ru nanosheets have variations of the number of layers. Assuming the thickness of 0.72 nm for 1 L from the difference between 1 L and the overlapped region (2 L), we obtained the variations for ±3.1 L for 6 L and ±3.0 for 9 L, respectively. The large variations of the few-layered Ru nanosheets suggest the acceleration of crystallization and/or the existence of the stacking fault by large motion of atom.

Photoemission spectroscopy measurements were performed at BL47XU of SPring-8, BL2A of Photon Factory (PF), and BL9A of Hiroshima Synchrotron Radiation Center (HiSOR) equipped the VG-Scienta R4000 electron analyzer. The electron analyzers are placed at 90° for SPring-8 BL47XU, 40° for PF BL2A, and 50° for HiSOR BL9A to the incident beam. The incident angles relative to the sample surface are set to at 12° for SPring-8 BL47XU, 50° for PF BL2A, and 40° for HiSOR BL9A. Linearly polarized photon of the incident beam was used at all beamlines. The beam sizes are about 40 *μ*m × 30 *μ*m for SPring-8 BL47XU, 100 *μ*m × 1  m for PF BL2A, and 1.5 mm × 1.0 mm^2^ for HiSOR BL9A. The photoemission data were collected at *T* = 300 K for *hv* = 7940 eV at SPring-8 BL47XU, *T* = 15 K for *hv* = 800 eV at PF BL-2A, and *T* = 28 K for *hv* = 20 eV at HiSOR BL-9A, respectively. By fitting of the gold spectrum to the Fermi-Dirac function convoluted with the Gaussian function corresponding to the total-energy resolution, we obtained the total-energy resolutions of 265 meV for *hv* = 7940 eV, 203 meV for *hv* = 800 eV, and 19 meV for *hv* = 20 eV, respectively. The base pressures of the spectrometer for each beamline were better than 1.0 × 10^−10^ Torr. The probability of a primary photoelectron escaping from the depth *z* is proportional to exp(−*z*/*λ*) where *λ* is an inelastic scattering mean free path (IMFP) for electrons in solid. Here, *λ* is about 7.4 nm for *hv* = 7940 eV, 1.3 nm for *hv* = 800 eV, and 1.0 nm for *hv* = 20 eV estimated from refs. ^10,11^.

The band structure calculation was carried out using the code WIEN2k^10^ based on the full-potential linearized augmented plane wave method. The calculated results were obtained the generalized gradient approximation for electron correlations, where we used the exchange-correlation potential^11^. The spin-orbit interaction is not taken into account.

## Results and Discussion

**Figure 2:**
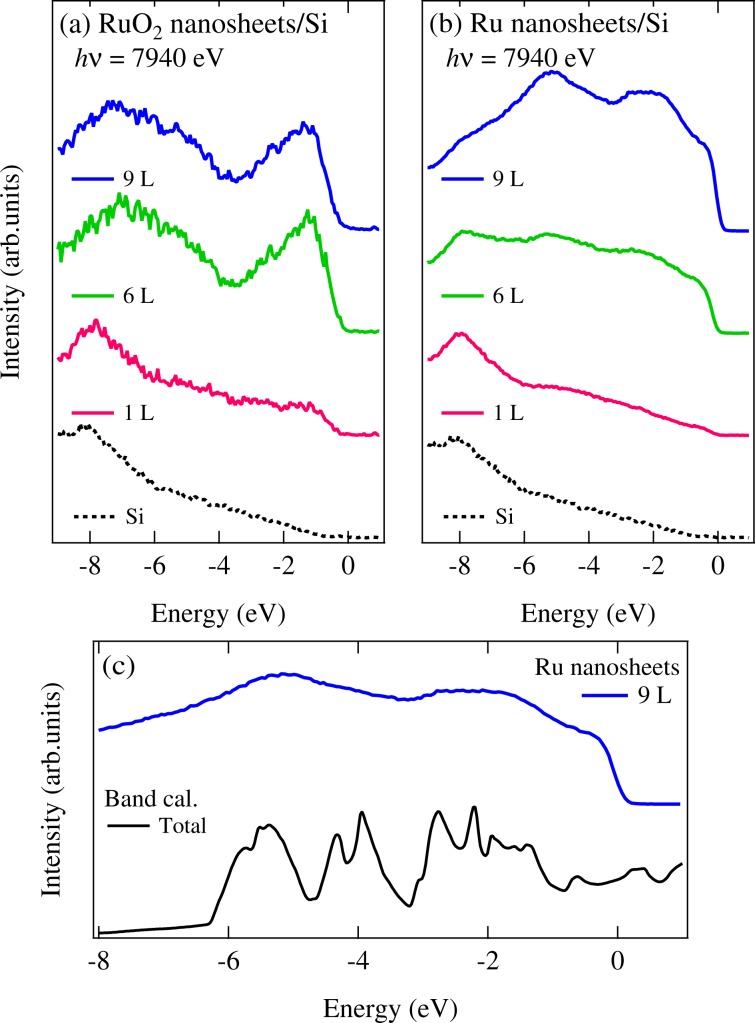
Valence-band photoemission spectra of (**a**) RuO_2_ and (**b**) Ru nanosheets (1, ~6, and ~9 L) taken at *hv* = 7940 eV. The dotted curve shows the spectrum of Si substrate without RuO_2_ and Ru nanosheets. (**c**) The valence band spectrum of the Ru nanosheets (~9 L) compared with the band structure calculation of bulk Ru metal. The data were collected at *T* = 300 K without surface treatment because of the large probing depth of hard x-ray. The spectra were normalized to the intensity around −8 eV.

Figure 2(a,b) show the valence-band photoemission spectra of RuO_2_ and Ru nanosheets (1, ~6, and ~9 L) on Si substrate. The dotted curve indicates the photoemission spectrum of Si substrate without RuO_2_ or Ru nanosheets. The intesity from the Si substrate as well as that from the nanosheets is certainly observed in RuO_2_ and Ru nanosheets, because of the large IMFP *λ* of 7.44 nm for the hard x-ray photons *hv* = 7940 eV^12^.

The spectral weights of RuO_2_ and Ru nanosheets increase as a function of the number of layers. In Fig. 2(c), we compare the valence band spectrum with the band structure calculation of bulk Ru metal obtained by using the WIEN2K package^10^. The observed structures in spectra are basically consistent with the band structure calculation. Meanwhile, for RuO_2_ nanosheets (1, ~6, and ~9 L), the spectral intensities around −1.5 eV and from −4 eV to −8 eV can be seen in Fig. 2(a). These features derive from the Ru 4*d* band and the O 2*p* band hybridized with the Ru 4*d* states, respectively. Furthermore, the spectral weight at *E*_F_ of RuO_2_ nanosheets is suppressed in contrast to that of the thick-layered Ru nanosheets. Thus, the spectral difference between the RuO_2_ and Ru nanosheets indicates that the Ru nanosheets are successfully elaborated from the RuO_2_ nanosheets by the hydrogen reduction method.

**Figure 3:**
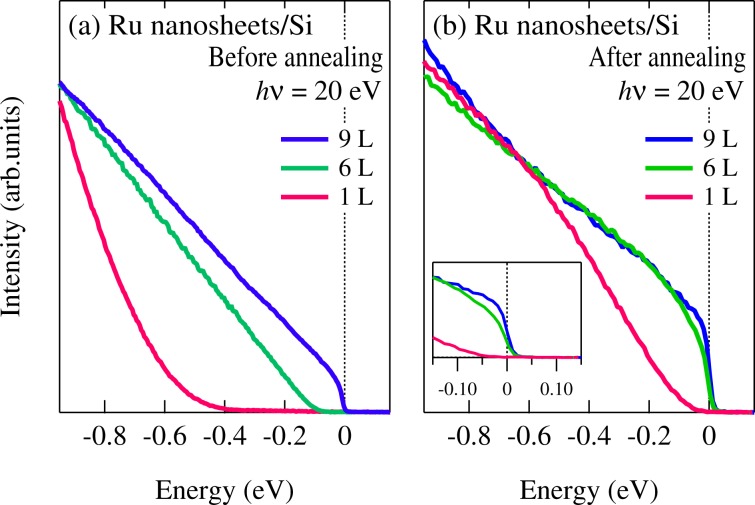
Near- *E*_F_ photoemission spectra of Ru nanosheets (1, ~6, and ~9 L) taken at *hv* = 20 eV (**a**) before annealing and (**b**) after annealing. Inset shows an enlarged view close to *E*_F_. The data were obtained at *T* = 28 K.

In order to discuss the conductivity from the electronic structure near *E*_F_, we performed the high resolution ultraviolet photoemission spectroscopy. Since the ultraviolet photoemission spectroscopy is the surface sensitive probe (*λ*~1.0 nm)^12^, we heated the Ru nanosheets up to 300 °C for 15–30 min to obtain the clean surface. Figure 3 shows the number-of-layers dependence of the near- *E*_F_ photoemission spectra before and after annealing. The near-*E*_F_ photoemission spectra before annealing exhibit the absence of the spectral weight at *E*_F_ even in the thick Ru nanosheets (~9 L), suggesting the contamination of the sample surfaces. After annealing, we observed the sharp Fermi cutoff in the spectrum of the thick Ru nanosheets (~9 L) and confirmed that the clean surfaces are obtained by annealing. However, the spectral weight at *E*_F_ of the mono-layered Ru nanosheets (1 L) is completely suppressed, indicating the non-metallic behavior as shown in the inset of Fig. 3(b). Interestingly, the spectral weight close to *E*_F_ for the thick Ru nanosheets of ~6 L diminishes in contrast to that of ~9 L.

**Figure 4:**
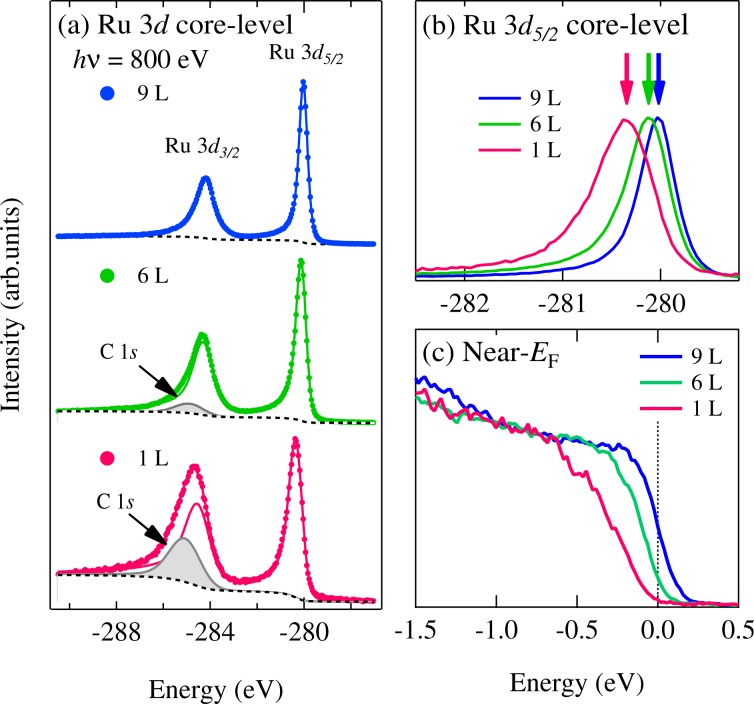
(**a**) Ru 3 *d* core-level spectra and (**b**) its enlarged plot of Ru nanosheets (1, ~6, and ~9 L). The photoemission intensity is normalized by the Ru 3 *d*_5/2_ peak intensity. (**c**) Near-*E*_F_ spectra of Ru nanosheets (1, ~6, and ~9 L). The spectra were taken at *hv* = 800 eV and *T* = 15 K. The samples were annealed *in situ* to obtain the clean surface.

In Fig. 4, the number of layers dependence of the photoemission spectra for Ru nanosheets (1, ~6, and ~9 L) are taken at *hv* = 800 eV. The samples were annealed to obtain the clean surface as the same processing in Fig. 3. Figure 4(a) shows the Ru 3 *d* core-level photoemission spectra of the mono- and few-layered Ru nanosheets (1, ~6, and ~9 L). Note that the C 1 *s* signal is completely absent in ~9 L sample, which indicates that clean surface was obtained by annealing process. On the other hand, the intensity of C 1 *s* starts to be observed from ~6 L and the intensity ratio of C 1 *s* to Ru 3 *d* core-levels increases in going from ~6 L to 1 L. This trend can be seen in the Si 2 *p*/2 *s* of the substrate and the O 2 *p* signals (see Supplementary Fig. S1). These suggest that the C 1*s* feature derives from the cationic coblock polyvinyl alcohol and polyvinyl amine polymers^7,9^ which mainly exist in between Ru nanosheets and Si substrate. Actually, the thickness of ~0.72 nm for 1 L is smaller than the escape depth *λ* = 1.22 nm for *hv* = 800 eV, consistent with the observation of the remarkable intensity of C 1 *s* in 1 L. In contrast, the thickness of ~4.32 nm for ~6 L is larger than *λ* and is correspond to 3 *λ*–4 *λ* (*λ* = 1.22 nm for *hv* = 800 eV), which explains the small intensity of C 1 *s* in ~6 L.

Figure 4(b) shows an enlarged view of the Ru 3 *d*_5/2_ core-levels. The peak positions of Ru 3 *d*_5/2_ core-level shift toward higher binding energy with decreasing the number of layers. This behavior is also observed in the previous photoemission study of the Ru nanosheets^9^. The observed shift of the Ru 3 *d* core-levels from ~9 L to 1 L results from the reduction of the screening effect corresponding to the number of conduction electrons. Indeed, the spectral weight near *E*_F_ decreases in going from ~9 L to 1 L and that of the mono-layered Ru nanosheets shows the insulating gap as shown in Fig. 4(c). Here, it should be noted that the peak width of Ru 3 *d*_5/2_ core-level becomes broad in going from ~9 L to 1 L. The Ru 3 *d* core-level broadening is probably due to the enhancement of disordered effect, which is reported in many alloys system such as Cu_1−*x*_ Pd_*x*_, Cu_1−*x*_ Pt_*x*_, and Pd_1−*x*_ Ag_*x*_^13–17^. Another possibility is that the increase of the Coulomb interaction induces the core-level broadening proposed by Davis and Feldkamp^18,19^.

**Figure 5:**
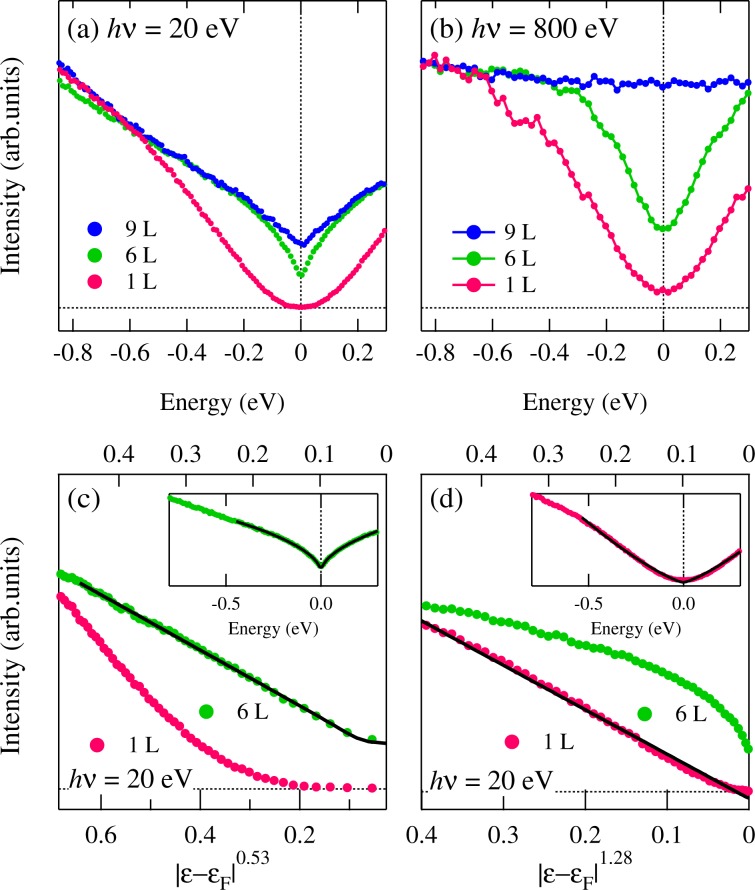
Symmetrized photoemission spectra of Ru nanosheets (1, ~6, and ~9 L) for (**a**) *hv* = 20 eV and (**b**) 800 eV. The data were taken at *T* = 28 K and *T* = 15 K, respectively. The samples were annealed *in situ* to obtain the clean surface. Symmetrized spectra in (**a**) are replotted as a function of (**c**) |*ε* − *ε*_*F*_|^0.53^ and (**d**) |*ε* − *ε*_*F*_|^1.28^. The insets indicate the symmetrized spectra and the fitted results. The solid curves represent the fitted curves.

Figure 5 shows the symmetrized near-*E*_F_ photoemission spectra of the Ru nanosheets (1, ~6, and ~9 L) taken at *hv* = 20 eV and 800 eV in order to visualize the spectral density of states near *E*_F_. The spectral density of states (DOS) of the mono-layer (1 L) and the thick layers (~6 L) depletes as it approaches *E*_F_ in contrast to that of ~9 L. The spectral DOS at *E*_F_ is completely suppressed in the mono-layered Ru nanosheets (1 L), while that of *N* = 6 L shows the cusp-like shape. These indicate the metal-insulator transition according to the thickness of Ru nanosheets. To address the mechanism of the metal-insulator transition of the Ru nanosheets, we simulate our data taken at *hv* = 20 eV using a power law *g*(*ε*) ∝ |*ε* − *ε*_*F*_|^*α*^ convoluted with the energy resolution 19 meV. The spectral DOS near *E*_F_ are well reproduced using the power factor *α* = 0.53 for 6 L and 1.28 for 1 L as indicated the solid curves in the insets of Fig. 5(c,d). In Fig. 5(c,d), we rescale the energy axis as a function of *α* = 0.53 and 1.28. The power factor *α* = 0.53 of ~6 L is comparable to a singularity of *α* = 0.5, which is estimated to consider the electron-electron interaction in three-dimensional disordered metals^20^ such as Ge_1−*x*_ Au_*x*_^21^. On the other hand, the energy dependence *α* = 1.28 in the mono-layered Ru nanosheets is close to *α* = 1. The power law with the exponent *α* = 1 indicates a soft Coulomb gap due to the disorder and the long-range Coulomb interaction, which is predicted in the 2-dimensional insulator by Efros and Shklovskii^22^. Therefore, our data suggest the transition from the metallic state of ~9 L to the 2-dimensional insulating state with the soft Coulomb gap of 1 L through the disordered 3-dimensional metallic state of ~6 L.

Here, we consider two main possibilities for the deviation from the power factor 1.0 to 1.28 in the mono-layered Ru nanosheets. On the basis of Efros-Shuklovskii theory, the spectral weight at *E*_F_ vanishes and the spectral weight near *E*_F_ follows the power law with the exponent *α* = 1.0 for the 2 dimensional system^22^. In the numerical calculation including the disorder effect and Coulomb interaction, the spectral weight near *E*_F_ is described by the power law with *α* = 1.2^23^. Moreover, the Coulomb gap is filled at finite temperature^24^ In any cases, the power factor *α* can be larger than 1.0. Alternatively, the deviation comes from the overlap region between the mono-layered Ru nanosheets. The AFM image of the mono-layered Ru nanosheets exhibits the overlap region between the Ru nanosheets. Since the electronic structure in the overlap region is not the 2-dimensional but the 3-dimensional, the power factor *α* would deviate from 1.0. Actually, we estimated the distribution of the mono-layered region and the overlap region from the AFM images as shown in Fig. 1. The distribution of the mono-layered region is 83.8% and that of the overlap region is 16.2%. On the basis of the Efros-Shuklovskii theory, the power factor *α* should be 2.0 for the 3-dimensional system. Considering the sum of the power factor *α* for each regions, the power factor *α* can be estimated *α* = 1.16.

Although the origin of enhancement of the disorder effects in ~6 and 1 L is still covered, the cusp-like spectral DOS near *E*_F_ is observed in ~6 L, which is a peculiar feature in the disordered metals. The increase of the disorder effect may induce the Ru 3 *d* core-level broadening as mentioned in Fig. 4. In addition to the disorder effect, the band width is reduced with decreasing the dimensionality. The Ru - Ru distance also extends from ~6 L to 1 L reported in the in-plane x-ray diffraction studies^9^. The resultant reduction of the band width would drive the enhancement of the effective Coulomb interaction in the mono-layered Ru nanosheets. Therefore, the disorder effect observed in ~6 L is more prominent in the mono-layered Ru nanosheets and the electron-electron correlation increases due to the band width narrowing from ~6 L to 1 L. In consequence, the interplay between the disorder and the Coulomb repulsion would form the soft Coulomb gap and the mono-layered Ru nanosheets become insulator. Our results suggest that the electric conductivity depending on the disorder and Coulomb interaction can be controlled by the number of stacking layers. Thus, the tunable electric conductivity using the Ru nanosheets will lead to the design of the multi-level resistance states for the electronic devices such as non-volatile storage devices. Here, the nanosheets in the present study have statistical dispersion of the number of layers. To further verify the precise dependence of the power factor on the number of layers and the critical thickness for the metal to insulator transition, it would be desirable to synthesize Ru nanosheets with more accurate thickness in future studies.

## Conclusion

In summary, we have performed the photoemission spectroscopy of the mono-layered and few-layered Ru nanosheets (1, ~6, and ~9 L) on Si substrate in order to reveal the electronic structure depending on the thickness of Ru nanosheets. The spectral DOS near *E*_F_ of 1 L and ~6 L depletes as it approaches *E*_F_, while that of ~9 L shows the clear Fermi cutoff. These are consistent with the chemical shifts of Ru 3 *d* core-level spectra. To verify the origin of the spectral weight suppression near *E*_F_ of ~6 L and 1 L, we reproduced the spectral DOS by using the power law. The power factor *α* increases in going from ~6 L to 1 L, indicating the enhancement of the disorder effect. We found that the transition from the metallic state of ~9 L to the 2-dimensional insulating state with the soft Coulomb gap of 1 L through the disordered 3-dimensional metallic state of 6 L.

## Supplementary Information

Supplementary Information.
