# Supplementary material for: Thickness-induced metal to insulator transition in Ru nanosheets probed by photoemission spectroscopy: Effects of disorder and Coulomb interaction
Source: Sci Rep. 2020 Jan 30;10:1541. doi: 10.1038/s41598-020-58057-9 (PMC6992751; doi:10.1038/s41598-020-58057-9)
Supplement: Supplementary file 1 — Supplementary Information. [file 41598_2020_58057_MOESM1_ESM.pdf]

Supplementary information

Thickness-induced metal to insulator transition in Ru nanosheets probed by photoemission spectroscopy : Effects of disorder and Coulomb interaction

Daiki Ootsuki[1*], Kenjiro Kodera[1], Daiya Shimonaka[1], Masashi Arita[2], Hirofumi Namatame[2], Masaki Taniguchi[2], Makoto Minohara[3], Koji Horiba[3], Hiroshi Kumigashira[3], Eiji Ikenaga[4], Akira Yasui[4], Yoshiharu Uchimoto[1], Satoshi Toyoda[5], Masahito Morita[6], Katsutoshi Fukuda[6], and Teppei Yoshida[1]

[1]*Graduate School of Human and Environmental Studies, Kyoto University, Sakyo-ku, Kyoto 606-8501, Japan.*

[2]*Hiroshima Synchrotron Radiation Center, Hiroshima University, Higashi-hiroshima 739-0046, Japan.*

[3]*Institute of Materials Structure Science, High Energy Accelerator Research Organization (KEK), Tsukuba, Ibaraki 305-0801, Japan. 4SPring-8/JASRI, 1-1-1 Koto, Sayo-cho, Hyogo 679-5198, Japan*

[5]*Department of Materials Science and Engineering, Kyoto University, Sakyo-ku, Kyoto 606-8501, Japan. 6Office of Society-Academia Collaboration for Innovation, Kyoto University, Sakyo-ku, Kyoto 606-8501, Japan.*

*ootsuki.daiki.4z@kyoto-u.ac.jp

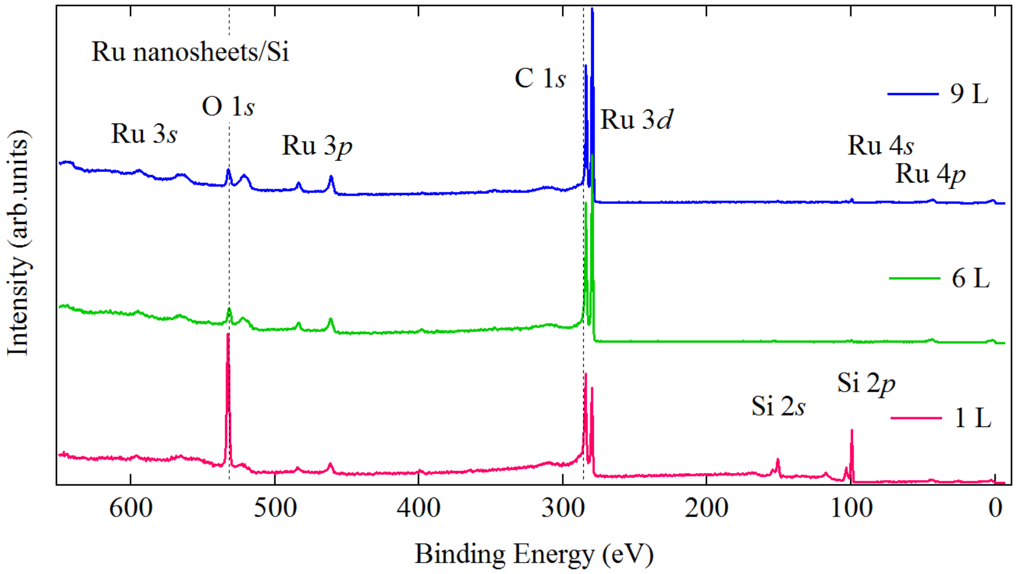

Figure S1. Survey scan photoemission spectra of the annealed Ru nanosheets (1 L, ~6 L, and ~9 L). The data were collected at $T = 50$ K and $h\nu = 800$ eV and normalized by the area.
